# Visualization and Pathological Characteristics of Hepatic Alveolar Echinococcosis with Synchrotron-based X-ray Phase Sensitive Micro-tomography

**DOI:** 10.1038/srep38085

**Published:** 2016-11-29

**Authors:** Huiqiang Liu, Xuewen Ji, Li Sun, Tiqiao Xiao, Honglan Xie, Yanan Fu, Yuan Zhao, Wenya Liu, Xueliang Zhang, Renyong Lin

**Affiliations:** 1 College of Medical Engineering and Technology, Xinjiang Medical University, Urumchi 830011, China; 2 Hepatobiliary & Echinococcosis Surgery, FirstAffiliated Hospital, Xinjiang Medical University, Urumchi 830054, China; 3 State Key Laboratory Incubation Base of Xinjiang Major Diseases Research, FirstAffiliated Hospital, Xinjiang Medical University, Urumchi 830054, China; 4 SSRF, Shanghai Institute of Applied Physics, Chinese Academy of Sciences, Shanghai 201800, China; 5 Imaging Center, First Affiliated Hospital, Xinjiang Medical University, Urumchi 830054, China

## Abstract

Propagation-based phase-contrast computed tomography (PPCT) utilizes highly sensitive phase-contrast technology applied to X-ray micro-tomography, especially with the extensive use of synchrotron radiation (SR). Performing phase retrieval (PR) on the acquired angular projections can enhance image contrast and enable quantitative imaging. We employed the combination of SR-PPCT and PR for the histopathological evaluation of hepatic alveolar echinococcosis (HAE) disease and demonstrated the validity and superiority of PR-based SR-PPCT. A high-resolution angular projection data set of a human postoperative specimen of HAE disease was acquired, which was processed by graded ethanol concentration fixation (GECF). The reconstructed images from both approaches, with the projection data directly used and preprocessed by PR for tomographic reconstruction, were compared in terms of the tissue contrast-to-noise ratio and density spatial resolution. The PR-based SR-PPCT was selected for microscale measurement and the 3D visualization of HAE disease. Our experimental results demonstrated that the PR-based SR-PPCT technique is greatly suitable for the discrimination of pathological tissues and the characterization of HAE. In addition, this new technique is superior to conventional hospital CT and microscopy for the three-dimensional, non-destructive microscale measurement of HAE. This PR-based SR-PPCT technique has great potential for in situmicroscale histopathological analysis and diagnosis, especially for applications involving soft tissues and organs.

Hepatic alveolar echinococcosis (HAE) is a rare disease caused by the intrahepatic development of the larvae of *Echinococcus multilocularis* tapeworms, which is a globally distributed zoonotic disease resulting in serious problems to the public health and national economy of many countries and regions worldwide, such as west China, Japan, Turkey, Eastern Russia, Central-Western Europe, and North America (Kern P *et al*.; Czermak B.V. *et al*.)^1^,^2^. Despite its parasitic nature, the HAE lesions of humans, similar to malignant tumours, are characterized by a high mortality rate and the ability to spread to other organs if no treatment is provided. Presently, the typical morphological imaging for the detection and pathological analysis of HAE in the hospital mainly depends on the following imaging techniques: (1) X-ray computed tomography (CT) based on conventional absorption-contrast/positron emission tomography combined with CT (PET/CT) are valuable but expensive and irradiative techniques in nuclear medicine for detecting disease metabolic activity (Caoduro C *et al*.; Reuter S *et al*.)^3^,^4^. (2) Ultrasound (US)/contrast-enhanced ultrasonography (CEUS) are the current methods of choice for the screening and monitoring of patients, systematically complemented by a CT-scan study (H.C. Zeng *et al*.; Piscaglia F. *et al*.)^5^,^6^. (3) Magnetic resonance imaging (MRI) is superior to assess the extent of the loco-regional disease and to characterize the different components of the lesions (Amel Azizi *et al*.; Coskun A *et al*.)^3^,^4^,^5^,^6^,^7^,^8^. These techniques of clinical imaging can usually achieve spatial resolutions of approximately one millimetre in exploring the fine structure of samples. Although electron microscopy, on the basis of micro-level sample slices, can achieve microscale or even quasi-nanoscale resolutions, it cannot meet the needs of 3D *in situ* or *in vivo* biomedical non-destruction studies (Laura Ceballos *et al*.)^8^,^9^. However, detailed three-dimensional, non-destructive microscale measurement is essential for the functional and pathological developmental study of soft biomedical samples. Moreover, accurate volumetric histopathological analysis of potentially affected tissue is crucial for the investigation and treatment of HAE diseases. X-ray computed micro-tomography (μ-CT) is a well-established technique for the three-dimensional imaging of the internal structure of objects. Conventional μ-CT is based on the differential attenuation of transmitted X-rays by structures within an object via a contrast mechanism that is effective for distinguishing between elemental components with significant differences in atomic number or electron density, such as bones or teeth. The difference in X-ray attenuation for different types of soft tissues is typically rather small, and this results in poor image contrast between soft biomedical fields and therefore hampers diagnostics and pathological analysis. However, the phase factor arising from the interaction with transmitting X-ray and soft tissues is approximately 10^3^ times higher than the corresponding absorption factor in the hard X-ray region^10^. This means that the phase contrast has a much higher sensitivity for soft tissues compared with simple absorption contrast. In addition, the current attenuation-based μ-CT technique is limited in its capability to improve its spatial resolution, in that the quantum noise variance is proportional to the fourth power of the resolvable spatial frequency with the standard filtered back projection (FBP) reconstruction algorithms. However, the noise scaling law for the phase-contrast μ-CT is completely different. Combining the phase retrieval and FBP algorithms, one can show the quantum noise variance scales linearly with the resolvable spatial frequency^11^,^12^,^13^,^14^. Hence the phase-contrast μ-CT technique has good potential for higher resolution tomography compared with attenuation-based μ-CT under the same contrast-to-noise ratio (CNR). It has been suggested that X-ray phase-contrast imaging can be utilized for improvement of the contrast in transmission images of soft biomedical samples consisting predominantly of light chemical elements.

Currently, advanced synchrotron radiation is featured as having higher brightness and coherence, compared with conventional X-ray tubes, and is superior for conducting research applications of synchrotron-based X-ray phase-contrast tomography (SR-XPCT), especially for various soft tissue pathological measurements. There are several SR-XPCT techniques that have emerged in recent decades based on the following methods: X-ray interferometry, analyser-based imaging (ABI), X-ray grating imaging (GI), and propagation-based imaging (PBI)^15^,^16^,^17^,^18^. Among the four micro-tomography techniques, the synchrotron-based X-ray phase-contrast micro-tomography (SR-PPCT) method–characterized as having the simplest experimental setup, minimized scanning time, and lower radiation dose–is suitable for the applied research of soft biomedical samples^19^,^20^,^21^,^22^,^23^. In this work, we aimed to apply the phase-retrieval-based SR-PPCT (PR-based SR-PPCT) technique to the pathological micro-structural measurement and analysis of HAE diseased tissues by performing imaging of HAE lesions from patient postoperative specimens to explore the microscopic HAE pathomechanism. Performing phase retrieval on acquired angular projections can enhance image contrast and enable the quantitative segmentation of pathological tissues without staining samples. It has great potential for providing new methods of HAE pathological analysis and revealing the role of HAE pathological development and the relationship between structure and function.

## Results

**Figure 1:**
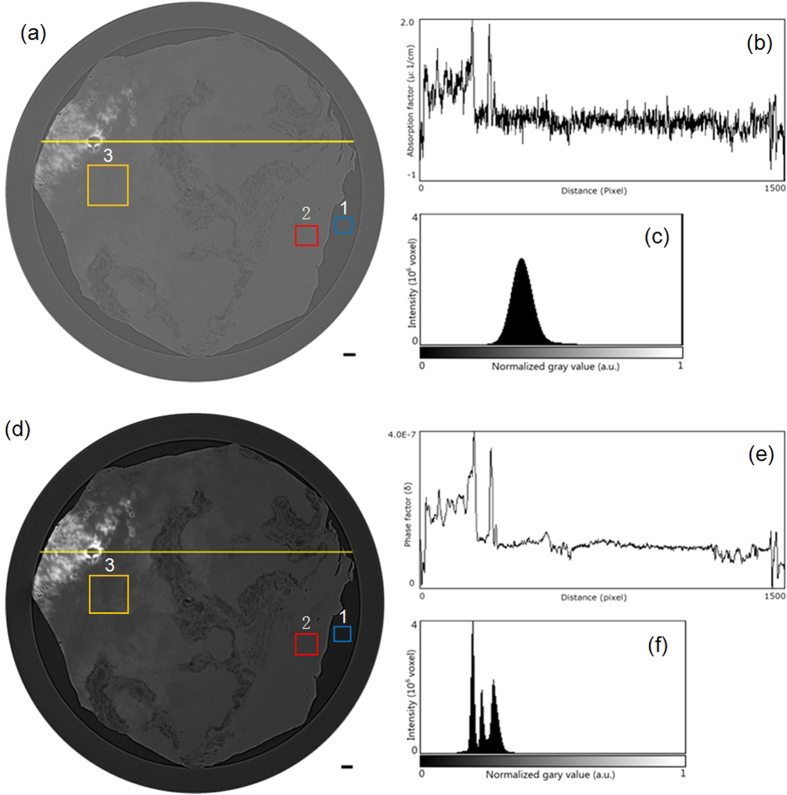
Reconstructed phase-contrast sectional images of a human HAE lesion with a slice thickness of 5.2 μm by using SR-PPCT (**a**) and PR-based SR-PPCT (**d**) methods. Profile lines, shown in (**b,e**), were plotted along the yellow solid lines in (**a,d**). Histograms measured in (**a**,**d**) are displayed in (**c,f**). The rectangle regions 1 and 2, plotted in (**a,d**), were used for calculating the CNRs of both PPCT techniques, and region 3 was used for enlarged views of higher resolution measurements. The length of the scale bar is 150 μm.

**Figure 2:**
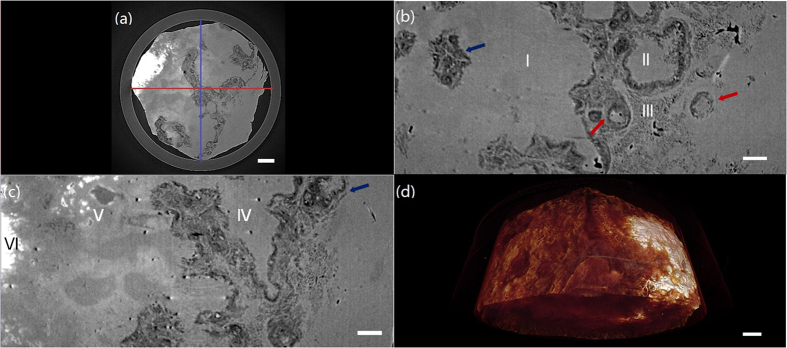
PR-based SR-PPCT sectional images of a human HAE lesion with a slice thickness of 5.2 μm. (**a**) Transversal section, (**b**) Sagittal section along the blue solid line in (**a,c**) Coronal section along the red solid line in (**a,d**) Three-dimensional rendering of the whole HAE specimen. Histopathological characteristic scan be clearly observed in (**b,c**), including liver parenchyma I, periphrastic granulomatous and fibrous tissues II, micro cysts III, small blood vessels IV, “wood wheel” sign (daughter cysts) V, completely calcified metamorphosis VI. The lengths of the scale bars are 300 μm in (**a,d**), 150 μm in (**b,c**).

**Figure 3:**
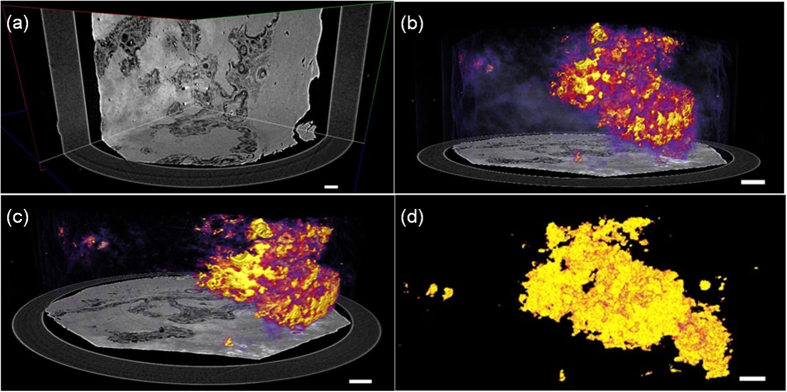
Three-dimensional renderings of PR-based SR-PPCT scans of different human HAE lesion components. (**a**) Virtual cut through the central volume of the HAE specimen in Fig. 4(d), showing the different HAE pathological feature structures and connectivity. (**b**) Delineation of the volumetric distribution of the HAE lesion, parenchyma and halo zone (marginal zone), surrounding the lesions, (**c**) shows the 3D morphology of fibrosis and calcification of lesion tissues, and (**d**) shows the volumetric distribution and 3D shape of the completely calcified lesions. The length of the scale bar is 150 μm.

**Figure 4:**
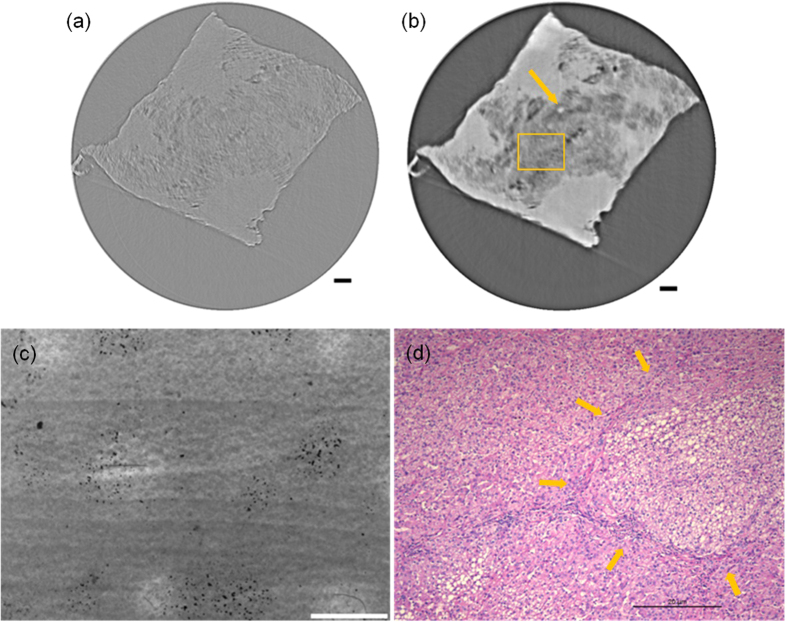
Comparison and evaluation of higher resolution SR-PPCT sectional image with a slice thickness of 1.625 μm and its histopathologic examination image of a local HAE lesion, corresponding to the rectangle region 3 in Fig. 1(a,d). Reconstructed sectional images using the SR-PPCT technique (**a**) and PR-based SR-PPCT technique (**b,c**) is the enlarged view of the rectangle plotted in (**b,d**) the H&E stained image under a light microscope (400×). The length of the scale bars are 80 μm in (**a,b**), and 20 μm in (**c,d**).

Figure 1(a,d) shows tomographic slices of the HAE patient with multiple lesions, based on the SR-PPCT experiment with an effective pixel size of 5.2 μm and makes a comparison between those with and without the PR algorithm under the same experimental condition. They both visualized the microscopic histopathological structural information, including the micro-calcification clusters, hepatic fibrosis distributions, micro blood vessels, etc., caused by *Echinococcus multilocularis*. Our results are consistent with the previous findings from optical microscopy of anatomic slices. Moreover, the tomographic slice with PR displayed a higher contrast and lower noise level than that without PR, as described with profile lines in Fig. 1(b,e). The electron density resolution of the PR slice is highly superior to that without PR, described with both histograms in Fig. 1(c,f), which easily enables the extraction of characteristic lesion areas. Figure 2 shows the phase-contrast sectional images and 3D rendering of the human HAE lesion from the transversal, coronal, and sagittal viewing angles, which can provide the *in situ* 3D histopathological structural information of a region of interest of HAE tissues at any perspective. In particular, the boundaries and interfaces of different HAE tissues can be distinguished mainly through the phase-contrast enhancements of the PR-based SR-PPCT technique. Figure 3(a) describes the 3D virtual cut through the central volumetric mass of Fig. 2(d), in which more detailed morphological visualization of pathological tissues at the micrometre scale is indicated clearly, especially for tiny cyst-like component lesions and micro-blood-vessels, which cannot be observed or are very blurred using the imaging modes utilized in hospitals. Figure 3(b–d) delineate the 3D rendered connection of different components of HAE lesions, corresponding to the volumetric distributions of “halo zones”, fibrotic tissues, and pure calcification areas, respectively, which reveal the main stages of the microscale formation of HAE. According to our findings, the early stage of HAE lesions may be located in the non-fibrosis and non-calcification areas, indicated by the orange solid-line rectangles 3 in Fig. 1(a,d), and may show weak contrast compared with the normal liver parenchyma. Therefore, we performed both higher resolution SR-PPCT techniques with an effective pixel size of 1.625 μm aiming at the local specimen in the rectangle 3 area, shown in Fig. 4(a) with SR-PPCT and (b) with PR-based SR-PPCT, which yielded different contrasts of tomographic results. Figure 4(c,d) are the enlarged views of the ROIs in Fig. 4(b). Moreover, Fig. 4(d) is the histopathological examinations with haematoxylin and eosin (H&E) staining under a light microscope. It can be observed that the outer layer of the HAE lesion in the early stage is characterized as the slightly fibrous tissue surrounded by the inflammatory response area with proliferation of small blood vessels.

## Discussion

The sectional images, reconstructed from both SR-PPCT and PR-based SR-PPCT techniques, were examined by the metrics of contrast-to-noise ratio (CNR), which can comprehensively evaluate the contrast enhancement and noise level of an image quantitatively. The following formula was used to calculate the CNR:


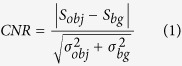


where *S* and *σ* represent mean pixel values and standard deviations, respectively, of a manually defined object and background regions of interest (ROI),subscripted by *bg* and *obj* and defined by the blue rectangle 1 and red rectangle 2 in Fig. 1(a,d). Their measured values of CNRs are 8.7 and 86.5, corresponding to the ROIs in Fig. 1(a,d), respectively. The PR-based SR-PPCT technique achieved almost 10 times higher CNR values than that with the SR-PPCT technique, which were also proven by their performance on noise level and density resolution, shown as profile lines in Fig. 1(b,c) and the histograms in Fig. 1(e,f). The data demonstrated that phase retrieval plays an important role in the visualization and quantitative segmentation of characteristic structural features in the histopathological analysis.

The results of our investigations presented different types of HAE lesions on the microscale level, characterized by the infiltration of micro vesicles in liver tissues. Accordingly, histopathological structures can be clearly observed, including the liver parenchyma (I), periphrastic granulomatous and fibrous tissues (II), micro cysts (III), small blood vessels (IV), “wood wheel” sign (daughter cysts) (V), and completely calcified metamorphosis (VI), shown in Fig. 2. There were enormous alveolar vesicles of different sizes and developmental stages in the tissue; thus, different pathologic features were identified as typical single vesicles (red arrows) and multiple vesicles (blue arrows), indicated in Fig. 2(b,c). Some were in the shape of branches, germinal layers with different thicknesses, exhibiting a punctual structure in the early developmental stage; some others contained substances in the fibrous tissues filled in the HAE cysts. Therefore, these results suggest that HAE lesions, differentiating into single or multiple vesicles, grew with various sizes and characteristics even in the same growth period, owing to differences in the host-immune reaction to *Echinococcus multilocularis*. In addition, the different enhancements of the HAE lesions, associated with different developmental stages, can generally be observed by the PR-based SR-PPCT technique, such as ring enhancements and central septa enhancements during the arterial and portal venous phases, exhibited in Fig. 2(b,c), identified as single or multiple vesicle structures with a surrounding inflammatory reaction belt and distribution of small blood vessels. In this study, without any staining process or contrast agents, the inflammatory reaction belt was obviously observed and confirmed what previous studies using contrast agent methods had reported (Asanuma *et al*.; Ehrhardt *et al*.; Guerret *et al*.)^24^,^25^,^26^. In Fig. 3(a), the clear 3D volumetric view of many inflammatory reaction belts revealed the proliferation of small blood vessel networks in these inflammatory reaction belts, displayed as many apparent dark spots of varying sizes. It has been suggested that the main blood supply of those smaller lesions resort to hepatic artery vessels (bigger dark spots); however, for the larger lesions, the portal veins (smaller dark spots) mainly take part in the blood supply. Actually, the inflammatory reaction belt is a product of the course of blocking the development of HAE lesions by the normal hepatic tissues, and the results in Fig. 3(b–d) showed the different statuses from the inflammatory reaction (lilac color) to periphrastic granulomatous and fibrous tissues (modena color), and eventually to completely calcified metamorphosis and coagulation necrosis (yellow color) in the view of three-dimensional transformation process, which corresponds to parasite degeneration. It represents the varying stages of HAE diseases, and the parasitic metabolic activity can lead to any inflammatory reaction derived from the viable vesicles, which would be responsible for the formation and development of microcysts gathered into “bunches of grapes” in the solid component regions of the HAE lesions. To discover additional and accurate pathological information of HAE lesions in early stage, corresponding to the area of rectangle 3 in Fig. 1, the higher resolution and contrast HAE measurement was performed by using PR-based SR-PPCT technique, shown in Fig. 4(b), is essential for the tiny size of an HAE lesion in an early stage, which is superior for the detection of small and weak HAE lesions compared with Fig. 4(a) using the SR-PPCT technique, due to the indistinct margins of a lesion and the blurred inflammatory reaction belts. For further comparison and verification with a pathologic examination, the H&E stained slices of the local HAE lesion, shown in Fig. 4(d), displayed a bright and round area with 20 μm diameter, consisting of clusters of microcysts smaller than 3 μm and surrounded by epithelioid cells, macrophages, fibroblast tissue, and lymphocytic infiltration, indicated by the arrows in Fig. 4(d). Thus, microcysts seem to be the sole reliable morphological indicator of disease activity, and the absence of microcysts could indicate degeneration. Therefore, the H&E stained slice corresponds to the characteristic spot, denoted by the arrow in Fig. 4(b), which is a typical single vesicle in the plasma (lipoid) at the early stage of pathology. In addition, Fig. 4(c) is the enlarged view of the green rectangle region in Fig. 4(b). The high resolution PR-based SR-PPCT technique has a higher sensitivity than that of the SR-PPCT technique in terms of distinguishing different types of HAE lesions, owing to the CNR-enhancement and better density resolution capability, especially for the evaluation of the correct treatment and early diagnosis based on pathology.

In conclusion, for three-dimensional, non-destructive high-resolution imaging tasks in medical histopathological analysis and diagnosis, we developed a synchrotron-based PPCT technique with phase retrieval to investigate HAE lesions and demonstrated the validity and advantages of the PR-based SR-PPCT technique. In our work, the experimental results showed a higher sensitivity in HAE lesion imaging, and demonstrated that this novel technique is superior for the three-dimensional discrimination and characterization of fine pathological structures on the microscale level, which is especially essential for the evaluation of the metabolic activity of HAE lesions. In comparison to conventional, high-resolution histopathological examination methodologies, such as TEM, SEM, and other microscopic techniques, this strategy with striking CNR-enhancement can provide non-invasive, microscale three-dimensional *in situ* pathological and morphological information, and facilitate the quantitative segmentation for characteristic structures, beyond the conventional resolution of CT, US, and MRI procedures available in hospitals. Hence, the PR-based SR-PPCT technique has a good potential for the histopathological measurement and analysis of HAE, and even for the medical pathological analysis of other diseases.

## Methods

### Data acquisition and processing

**Figure 5:**
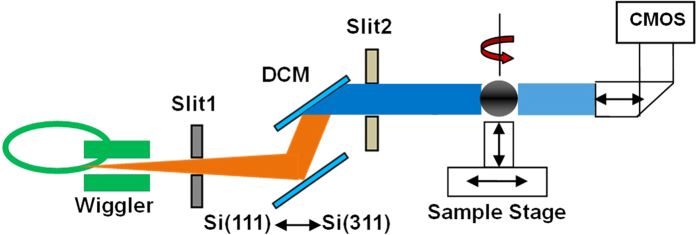
Schematic of SR-PPCT experimental setup at SSRF.

The synchrotron-based biomedical experiment was performed in the X-ray Imaging and Biomedical Application Beamline (BL13W1) at the Shanghai Synchrotron Radiation Facility (SSRF, China). The experimental setup of propagation-based phase-contrast computed tomography with synchrotron radiation (SR-PPCT) is depicted in Fig. 5.

The X-ray beam is produced by a 16-pole wiggler source on a 3.5 GeV storage ring (with ring current I = 250 mA, top-up mode) and monochromated with a double-crystal monochromators (DCM) tuning into the energy range from 8 to 72.5 keV, which can provide not only the appropriate spatial coherence but also a nearly parallel beam incident on a sample, placed approximately 34 m downstream from the wiggler source. Through adjusting the sizes of slit1 and slit2, the maximal beam size can reach 48 mm (horizontal) and 5 mm (vertical) with a divergence of 1.5 mrad (horizontal) and 0.2 mrad (vertical). The KOHZU six-dimensional sample stage is fixed on the 3-meter-long granite experimental platform installed at the end-station and intended for high stability and high-resolution imaging. Coupled with a 100 μm thick phosphor screen and visible-light optics for yielding various effective-pixel-sizes, the CMOS detector (C11440–22C, Hamamatsu Photonics, Japan) with the original pixel size of 6.5 μm was installed on a precise slide rail along with remote control motors capable of providing an easily adjustable sample-to-detector distance (SDD). In our biomedical experiment based on the SR-PPCT technique, the data sets were acquired at SDD = 180 mm and with the X-ray energy of 15 keV by tuning DCM Si (111),the row images consisted of 1080 tomographic projections over 180° of rotation, the X-ray phase-contrast angular projections were collected using the IMAGE PRO Plus 6.0 software package. In general, angular projections are performed first at a given SDD by using the propagation-based phase contrast technique. From these angular projections, three-dimensional tomograms are then reconstructed using the standard filtered back-projection (FBP) algorithm. In each of the original angular projections, the phase-contrast mechanism generates an absorption contrast of bulk uniform tissues and edge-enhancing interference fringes at the interfaces of different tissues, and thus the reconstructed SR-PPCT tomograms of soft tissues exhibit weakly absorbed bulk contrast and edge-enhancement at the tissue interfaces and boundaries^27^,^28^,^29^,^30^. Actually, these tomograms cannot satisfy the needs of analysis of quantitative characterizations for soft tissues or materials. The phase shift-induced interaction of the X-rays and material is over 1000 times larger than that of the absorption coefficient for the hard X-rays, and therefore the processing of phase retrieval (PR), characterized by striking contrast enhancement and lower noise level, is very necessary for medical pathological examination and quantitative analysis, especially for facilitating the characteristic visualization and segmentation based on the phase contrast. Hence, the PR-based SR-PPCT technique was employed to conduct this investigation of HAE lesions, which can be performed with a two-stage process: First, the acquired projections are pre-processed with PR algorithms. There are several kinds of phase retrieval method in the recent decade^31^,^32^,^33^,^34^,^35^, and we adopted the single-distance and steady PR algorithm in our experiment, presented by D. Paganin *et al*.^36^. For a weak absorption and quasi-homogeneous sample, the real and imaginary parts (correspondingly denoted as *δ* and *β*) of its complex refraction index are proportional to each other, and the ratio *δ/β* is a constant for a particular sample. This ratio for a sample can be estimated from the X-ray databases (CXRO,2012; Henke *et al*.^37^; NIST database)^37^. Therefore, the phase retrieval formula can be written as the following:


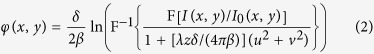


Here, 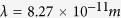 is X-ray wavelength, x and y are the Cartesian coordinates of the image and/or sample plane, u, v are the Fourier conjugate coordinates of x and y, and z is the SDD = 180 mm. F and F^−1^ are the forward and backward Fourier transform operators, respectively. 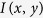 denotes the intensity distribution in the phase-contrast radiograph, 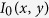 is the incident intensity without the sample at the position of the detector. The value of *ε = δ/β* was set to 1000 in our reconstruction. Second, three-dimensional images were reconstructed with the standard FBP algorithm. Prior to PR and image reconstruction, dark signal correction and flat field normalization were both applied to each raw dataset.

### Specimens

**Figure 6:**
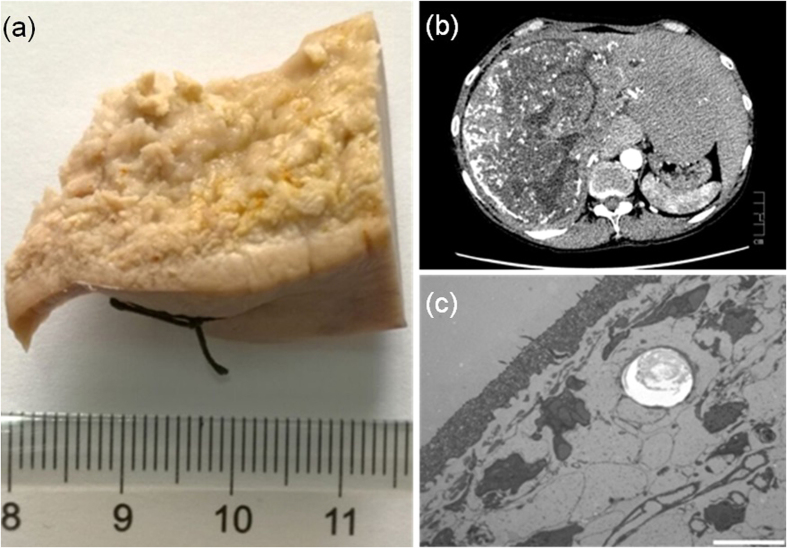
Human HAE disease specimen used in our experiment and the examination results in the hospital. (**a**) HAE specimen after GECF process. (**b**) Hospital CT slice with a centimetre ruler. (**c**) TEM slice with the scalebar of 1 μm and cutting thickness of 1 μm.

To demonstrate the validation and superiority of medical histopathological research with PR-based SR-PPCT, our measurements utilized surgical pathologically confirmed specimens of an HAE patient (female, 43 years old) with a confirmed HAE diagnosis based on X-ray radiology and CT in the hospital, shown in Fig. 6(a,b). In addition, the TEM result of the stained and 1 μm thickness slice of this postoperative specimen is shown in Fig. 6(c). This study was approved by our clinical ethics committee of the First Affiliated Hospital of Xinjiang Medical University, and the requirement for patient informed consent was waived. The criteria to be included in our study were patients recorded in the Registry database, with serological criteria in accordance with World Health Organization-Informal Working Group on Echinococcosis recommendations. The *ex vivo* HAE sample without staining was maintained in a formalin solution (10% formalin neutral buffer solution) at room temperature. A process of graded dehydration series of ethanol solution (short: graded ethanol concentration fixation-GECF) was employed 48 hours before our experiments, and the sample was placed in an upright position in a plastic tube to facilitate the propagation-based experimental setup.

## Additional Information

**How to cite this article**: Liu, H. *et al*. Visualization and pathological characteristics of hepatic alveolar echinococcosis with Synchrotron-based X-ray Phase Sensitive Micro-tomography. *Sci. Rep*. **6**, 38085; doi: 10.1038/srep38085 (2016).

**Publisher's note:** Springer Nature remains neutral with regard to jurisdictional claims in published maps and institutional affiliations.
